# Peer Victimization and Dysfunctional Reward Processing: ERP and Behavioral Responses to Social and Monetary Rewards

**DOI:** 10.3389/fnbeh.2019.00120

**Published:** 2019-05-31

**Authors:** Brent I. Rappaport, Laura Hennefield, Autumn Kujawa, Kodi B. Arfer, Danielle Kelly, Emily S. Kappenman, Joan L. Luby, Deanna M. Barch

**Affiliations:** ^1^Department of Psychological & Brain Sciences, Washington University in St. Louis, St. Louis, MO, United States; ^2^Department of Psychiatry, School of Medicine, Washington University in St. Louis, St. Louis, MO, United States; ^3^Department of Psychology & Human Development, Vanderbilt University, Nashville, TN, United States; ^4^Center for HIV Identification, Prevention, and Treatment Services, University of California, Los Angeles, Los Angeles, CA, United States; ^5^Department of Psychology, San Diego State University, San Diego, CA, United States; ^6^Department of Radiology, School of Medicine, Washington University in St. Louis, St. Louis, MO, United States

**Keywords:** peer victimization, event-related potentials (ERP), reward, depression, adolescence, monetary reward, social reward

## Abstract

Peer victimization (or bullying) is a known risk factor for depression, especially among youth. However, the mechanisms connecting victimization experience to depression symptoms remains unknown. As depression is known to be associated with neural blunting to monetary rewards, aberrant responsiveness to social rewards may be a key deficit connecting socially stressful experiences with later depression. We, therefore, sought to determine whether adolescents’ experiences with social stress would be related to their current response to social rewards over less socially relevant monetary rewards. Neural responses to monetary and social rewards were measured using event-related potentials (ERPs) to peer acceptance and rejection feedback (Island Getaway task) and to monetary reward and loss feedback (Doors task) in a sample of 56 late adolescents/emerging young adults followed longitudinally since preschool. In the Island Getaway task, participants voted whether to “keep” or “kick out” each co-player, providing an index of prosocial behavior, and then received feedback about how each player voted for the participant. Analyses tested whether early and recent peer victimization was related to response to rewards (peer acceptance or monetary gains), residualized for response to losses (peer rejection or monetary losses) using the reward positivity (RewP) component. Findings indicated that both experiencing greater early and greater recent peer victimization were significantly associated with participants casting fewer votes to keep other adolescents (“Keep” votes) and that greater early peer victimization was associated with reduced neural response to peer acceptance. Early and recent peer victimization were significantly more associated with neural response to social than monetary rewards. Together, these findings suggest that socially injurious experiences such as peer victimization, especially those occurring early in childhood, relate to two distinct but important findings: that early victimization is associated with later reduced response to peer acceptance, and is associated with later tendency to reject peers. Findings also suggest that there is evidence of specificity to reward processing of different types; thus, future research should expand studies of reward processing beyond monetary rewards to account for the possibility that individual differences may be related to other, more relevant, reward types.

## Introduction

Peer victimization (i.e., bullying) affects nearly one-fifth of high school students in the United States and over a third of adolescents worldwide (Modecki et al., 2014; US Center for Disease Control, 2018) and is an established risk factor for psychopathology. More specifically, victimized youth have a heightened risk for depression (Reijntjes et al., 2010; Takizawa et al., 2014; Klomek et al., 2015). Depression is associated with blunted neural responses to rewarding feedback in adults and adolescents (for meta-analyses, see Zhang et al., 2013; Keren et al., 2018). Though functional magnetic resonance imaging (fMRI) studies initially focused on hyporeactivity to monetary rewards, recent studies have extended the findings to *social* rewards (Olino et al., 2015; Kujawa et al., 2017), suggesting that depression is associated with anhedonia to multiple different reward types (Fussner et al., 2018). Some have proposed that this anhedonia is the result of interactions between the reward system and stress (Pizzagalli, 2014), showing that acute stress reduces striatal activation to monetary rewards (Ossewaarde et al., 2011; Porcelli et al., 2012). Therefore, peer victimization may lead to blunting of the brain’s response to rewards. As a social experience, peer victimization might be expected to be more strongly related to aberrant responses to social rewards than monetary ones. This is because victimization could change the value associated with positive peer feedback, making youth glean less pleasure or sense of reward from social acceptance. On the other hand, peer victimization may be related to depression just as any other childhood (Mandelli et al., 2015) or lifetime stressor (Kendler et al., 1999), with the social component of the stressor irrelevant. If so, peer victimization may act similarly to other childhood stressors in contributing to risk for depression, and thus may be related to blunted responses to both monetary and social rewards. Either pattern of responses would inform the pathway through which victimization confers risk for depression. Identifying this pathway can lead to interventions aimed at preventing or reducing the occurrence of depression in victimization youth. As such, the goal of the current study was to determine whether peer victimization was similarly or differentially associated with brain response to social and monetary rewards in the same sample.

One measure of reward response studied in depression is reward-related activity, occurring in response to the presentation of reward feedback. This can be measured using event-related potentials (ERPs)—an EEG signal time-locked to a particular event, such as the onset of a stimulus. ERP signals consist of components related to specific cognitive, motor, sensory, or emotional processes (Luck and Kappenman, 2012), including the reward positivity (RewP), an ERP component related to the processing of rewarding feedback. The RewP is thought to arise from activity within the mesocorticolimbic circuit including the striatum, mPFC, amygdala and orbitofrontal cortex (Gehring and Willoughby, 2002; Carlson et al., 2011; Foti et al., 2011b; Becker et al., 2014; Weinberg et al., 2014; Proudfit, 2015). Blunted RewP to monetary rewards has been concurrently and prospectively associated with depression severity in patients (Foti et al., 2011a, 2014; Bress et al., 2012; Liu et al., 2014; Proudfit, 2015), in some cases predicting risk for later depression (Bress et al., 2013; Weinberg et al., 2014, 2015; Nelson et al., 2016). More recently, depression severity has been associated with blunted RewP to social rewards (Kujawa et al., 2017), with one study directly comparing RewP to monetary and social rewards and revealing morphologically similar, although not identical, waveforms of activation (Ethridge et al., 2017). This makes the RewP an interesting and well-validated ERP component to test whether peer victimization is similarly or differentially associated with aberrant brain responses to rewards of different types.

In addition to neural responses, prosocial behavior towards peers may also inform our understanding of the link between peer victimization and depression. For instance, social acceptance is related to more prosocial behavior (Tur-Porcar et al., 2018; Will et al., 2018) and prosocial behavior itself is associated with improved social acceptance and relationships (Crick, 1996; Layous et al., 2012). In contrast, social rejection is linked to more aggressive and less prosocial behavior (Di Giunta et al., 2018; Tur-Porcar et al., 2018) in addition to causing a reduction in prosocial behaviors such as donating money, volunteering, helpfulness, and cooperation (Twenge et al., 2007). These findings suggest that the way youth react behaviorally to negative social interactions could reduce their ability or motivation for positive engagement and further deteriorate their peer relationships, overtime worsening depression symptoms (Leadbeater and Hoglund, 2009). Thus, while it is important to examine potential neural mechanisms of risk, behavioral mechanisms likely contribute to the relationship between peer victimization and depression. To test this, the current study also assessed whether peer victimization was associated with reduced prosocial behavior towards other co-players during the social reward task.

There is a reason to believe that both recent and early life experiences with peer victimization could be associated with aberrant reward responding. Recent, acute experiences of peer victimization affect adolescents’ schemas of peers and bias their interpersonal skills and attributions of peers (Schwartz et al., 1998; Camodeca and Goossens, 2005; Troop-Gordon and Ladd, 2005; Hoglund and Leadbeater, 2007). Despite peer victimization research tending to focus on adolescence, there is evidence that peer relations are as complex and salient in preschool (Schaefer et al., 2010), and that peer victimization is moderately stable beginning in early childhood (Pouwels et al., 2016). Thus, peer victimization experienced early in life may similarly bias individuals’ beliefs about others. This, in turn, could have long-lasting consequences for how victimized youth process and interpret social feedback, including peer acceptance and, subsequently, how their brain’s reward system develops and responds to social rewards. In fact, early social stress has been shown to lead to reduced behavioral reward learning (Guyer et al., 2006; Sheridan et al., 2018) and neural responses to reward (Hanson et al., 2016). This line of reasoning suggests that both recent and early experiences of peer victimization are relevant to the development of neural and behavioral reactions to rewards—including social rewards. While few studies have tested for a relationship between peer victimization and reward functioning (but see Casement et al., 2014; Ethridge et al., 2018), fewer still have included measures of peer victimization in early childhood. However, one study demonstrated that peer victimization can have long-lasting associations with responses to monetary reward, showing that greater victimization in late childhood predicted blunted brain responses to reward at age 16 (Casement et al., 2014). Another study found that early experience of peer victimization resulted in increased neural responsivity to social rejection in adolescence (Rudolph et al., 2016). Thus, there is intriguing evidence supporting the possibility that peer victimization in early childhood has lasting effects on adolescents’ responses to reward-related feedback; however, no study thus far has compared the relative strength of these associations between monetary and social rewards.

Given the literature reviewed above, the current study sought to examine the relationship between experiences of both early and recent peer victimization and current neural responses to social rewards (i.e., peer acceptance and rejection) compared to monetary rewards (i.e., gains and losses) in adolescents participating as part of a longitudinal study on early onset depression. We used two tasks to assess ERP responsivity to rewards: the Doors task was used to measure responses to monetary gains and losses, and the Island Getaway task was used to measure responses to social acceptance and rejection. Both tasks have been shown to elicit the Reward Positivity component (i.e., RewP). Behavioral responses on the Island Getaway task included voting to accept or reject other co-playing peers during the task. We tested the prediction that early and recent peer victimization would be more strongly related to blunted brain responses to social acceptance than to monetary gains. We also hypothesized that early and recent peer victimization would be related to less prosocial (acceptance) voting behavior. Finally, we tested the prediction that greater current depression symptoms would be related to reduced RewP responses in both tasks.

## Materials and Methods

### Participants

Participants were drawn from the Preschool Depression Study (PDS), a prospective longitudinal investigation of young children and their families conducted at a midwestern university in the United States (Luby et al., 2009). Details of recruitment have been previously reported (Luby et al., 2009, 2014). To briefly summarize, 3- to 6-year-olds were recruited from primary care practices and preschools/daycares throughout the St. Louis metropolitan region using a validated screening checklist [Preschool Feelings Checklist (Luby et al., 2004)] to oversample preschoolers with symptoms of depression and healthy controls. Parental written consent and child assent were obtained before participation and the local Institutional Review Board approved all procedures. These children have participated in up to 10 in-person clinical and behavior assessment and five neuroimaging assessments. In the most recent wave of data collection, a task measuring ERP responses to social feedback was added. The current study reports on 56 adolescents (46% female, mean age = 18.05 ± 1.01, 57% Caucasian, 34% African American, 9% Other) from the PDS who had completed the current wave of the study, with data collection ongoing. Of those, 16 participants had current clinical diagnoses of major depressive disorder (MDD) and 13 of MDD not otherwise specified. Of the 56 participants, 13 reported taking psychotropic medications in the past year.

### Measures

#### Social Reward Task

The Island Getaway task (Kujawa et al., 2014, 2017; Ethridge et al., 2017; Ethridge and Weinberg, 2018) was used to assess ERP and behavioral responses to peer acceptance or rejection. The original task was slightly modified to be age appropriate for the current sample. Task code is available at: http://arfer.net/projects/survivor. In the task, participants are told they are playing a game with real peers during which they will vote whether they wanted each peer (i.e., co-player) to continue on with them in the game, and then received feedback on how each co-player voted for them. Trials were divided into six rounds of voting. In the first round, participants created a profile including their photograph and demographic information and reviewed profiles of computerized co-players. In subsequent rounds, participants first responded to a poll question (e.g., “Who do you most admire?”) and then reviewed co-player responses in order to facilitate an exchange of personal information for the remaining voting and feedback phases.

**Figure 1:**
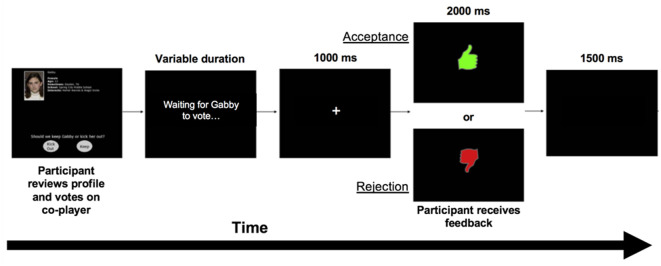
Voting and feedback trial during the Island Getaway task.

After reviewing each co-player’s profile and poll response in each round, participants completed a voting and feedback phase during which they voted to either accept (“Keep”) or reject (“Kick out”) each co-player, and after each vote received feedback indicating whether that co-player had voted to accept or reject them. Acceptance feedback was indicated by an image of a green “thumbs up” and rejection feedback was indicated by a red “thumbs down.” Each voting trial began with a co-player’s profile presented until participants voted. To simulate variation in co-player response speed, co-player voting time was selected for each trial based on actual variability in participants’ voting speeds from previously collected data. If participants voted faster than the simulated voting time for that co-player, the message “Waiting for [co-player’s name] to vote‥.” was displayed. Lastly, a fixation cross was presented for 1,000 ms, followed by feedback displayed for 2,000 ms. A blank screen was presented for 1,500 ms before the start of the next trial (see Figure 1).

Co-players were randomly assigned a voting pattern for each participant, such that two co-players rejected the participant on most (four or five out of six) rounds, two co-players accepted the participant on most rounds, and the remaining seven co-players were equally likely to accept or reject the participant. To increase the unpredictability of feedback, all co-players voted both to keep and kick out the participant at least once (with the exception of the co-player excluded after the first round). After each of the rounds, participants were told which one of the co-players had been voted out of the game. The task included a total of 51 feedback trials split evenly between acceptance and rejection, with the last trial type determined randomly, though the proportion of rejection and acceptance feedback in each round varied slightly across participants.

#### Monetary Reward Task

The Doors Guessing Task (see Supplementary Figure S1) has been used in previous studies of older children, adolescents, and adults with depression (Foti et al., 2011a,b, 2014; Bress et al., 2012, 2015; Nelson et al., 2015). Participants were shown a graphic displaying two adjacent doors and told to select a door to win $0.50 or lose $0.25. Following each choice, a feedback stimulus (green up arrow or red down arrow) appeared on the screen informing the children whether they lost or gained money. The order and timing of all stimuli were as follows (see Supplementary Figure S1): (i) the text “Click for the next round” was presented until the participant pressed a button; (ii) a fixation cross was presented for 1,000 ms; (iii) the graphic of two doors was presented until a choice was made; (iv) a fixation cross was presented for 1,000 ms; (v) a feedback arrow was presented for 2,000 ms, and finally; (vi) a fixation cross was presented for 1,500 ms. A green upward arrow indicated a correct guess and a red downward arrow indicated an incorrect guess. Participants received negative feedback on exactly 50% of the trials, and positive feedback on exactly 50% of the trials.

Recent evidence supports the psychometric properties of the Island Getaway and Doors tasks, including internal consistency and convergent validity between the tasks (Levinson et al., 2017; Ethridge and Weinberg, 2018).

#### EEG Data Collection and Processing

Continuous EEG was recorded using the BrainVision ActiChamp, 32 channel active channel amplifier system (BrainVision LLC, Morrisville, NC, USA). The electrodes were mounted in an elastic cap using a subset of the International 10/20 System sites (FP1, F3, F7, FC1, FC5, FT9, C3, T7, CP1, CP5, TP9, P3, P7, O1, Fz, Cz, Pz, Oz, FP2, F4, F8, FC2, FC6, FT10, C4, T8, CP2, CP6, P4, P8, TP10, O2) with a ground electrode located at FPz. The electrooculogram (EOG) generated from blinks and eye movements were recorded from five facial electrodes placed around the eyes. The EEG was sampled at 500 Hz and all signals were digitized on a laboratory computer.

#### Depression Symptoms

Current depression symptoms were measured as the sum of core symptoms of MDD endorsed by a clinician on the Kiddie Schedule for Affective Disorders and Schizophrenia (KSADS) at the current wave. Current depression symptoms were additionally measured using self-reported scores on the Child Depression Inventory–2 (CDI) if the participant was under 18 years old and Beck Depression Inventory–II (BDI) if the participant was 18 years old or older. CDI/BDI scores were calculated as the percentage of the raw score out of the total possible score, so as to make scores between the CDI and BDI comparable. Of the 56 participants, one was missing a CDI/BDI score. No participants were missing a score of core symptoms of MDD on the KSADS. Neural responses to monetary and social reward, as well as voting behavior, did not significantly differ from participants with missing CDI/BDI scores. Internal consistency was good for both CDI and BDI (Cronbach’s alpha = 0.91 and 0.82, respectively).

#### Measures of Peer Victimization

Peer victimization was measured using the Global Peer Relations scale of the Health and Behavior Questionnaire (HBQ; Armstrong and Goldstein, 2003). This scale includes items assessing peer acceptance/rejection and physical victimization, as well as relational victimization for children years old or older. Parents completed the child version (1.0) of the HBQ when children were 8 years old or younger, and the teen version (2.1) of the HBQ when children were 9 years old or older. Early experience of peer victimization was measured as the average score on this scale from the first three assessment waves, and recent experience of peer victimization was measured as the score on this scale from the previous wave (age range = 14.35–17.83 years). Internal consistency was good for the HBQ at early and recent assessment waves (Cronbach’s alphas = 0.84–0.91). Of the 56 participants, two were missing a measure of early peer victimization, and one was missing a measure of recent peer victimization. Neural responses to monetary and social reward, as well as voting behavior, did not significantly differ from participants with missing peer victimization scores. The results for subtypes of peer victimization (i.e., physical victimization, rejection, and relational victimization) are presented in the Supplemental Materials.

### Data Analysis

Off-line analysis was conducted using Brain Vision Analyzer 2 software (Brain Products, Gilching, Germany) and all data were re-referenced to the average of Tp9, Tp10, and Cz and band-pass filtered from 0.1 to 30 Hz. The EEG was corrected for EOG artifacts (Gratton et al., 1983) and physiological artifacts removed using an automatic procedure with a maximum allowed voltage step of 50 μv within a 400 ms interval length, maximum absolute different between any two points of 175 μv, and a minimum allowed activity of 0.50 μv within a 100 ms interval length. For both tasks, the EEG was segmented into 1,000 ms epochs, beginning 200 ms before and ending 800 ms after feedback onset. ERPs were quantified separately for the acceptance/gain and rejection/loss conditions as the mean activity at the Cz electrode site from 250 to 350 ms after feedback presentation in the Doors task and from 275 to 375 ms after feedback presentation in the Island Getaway task. This scoring is based on prior research showing that RewP is maximal in this time-frame and at this electrode for both tasks (Ethridge et al., 2017; Kujawa et al., 2017); of note, a study of the RewP response to monetary and social rewards in these two tasks found no difference in the psychometric properties of the RewP at Cz vs. frontal electrodes (i.e., Fz, FC1, FC2; Ethridge and Weinberg, 2018). A later time window is used for the Island Getaway task following studies that used principal component analysis to show that the RewP peaks approximately 25 ms later to social than monetary feedback (Ethridge et al., 2017; Kujawa et al., 2017; Babinski et al., 2019). Results were consistent when mean activity from 250 to 350 ms was used for the Island Getaway task (see Supplementary Materials). In line with previous work and recommendations (Meyer et al., 2017), residual scores for the RewP response to acceptance/gain accounting for RewP response to rejection/loss were calculated in R (version 3.5.0; R Core Team, 2013) to produce a score that was uncorrelated with RewP response to rejection/loss feedback. Residualized scores such as these are used to identify activity specific to reward response and account for other overlapping processes present in the ERP signal but unrelated to reward response (Luck and Kappenman, 2012). To test for associations between peer victimization and depression symptoms and brain and behavioral responses, robust linear regressions were fit using an M estimator from the MASS package (Venables and Ripley, 2002), and a robust *f*-test (Wald test) computed using the sfsmisc package (Maechler, 2018). *Z* tests were used to compare the regression coefficients of peer victimization predicting brain responses to social and monetary rewards (Paternoster et al., 1998).

## Results

**Figure 2:**
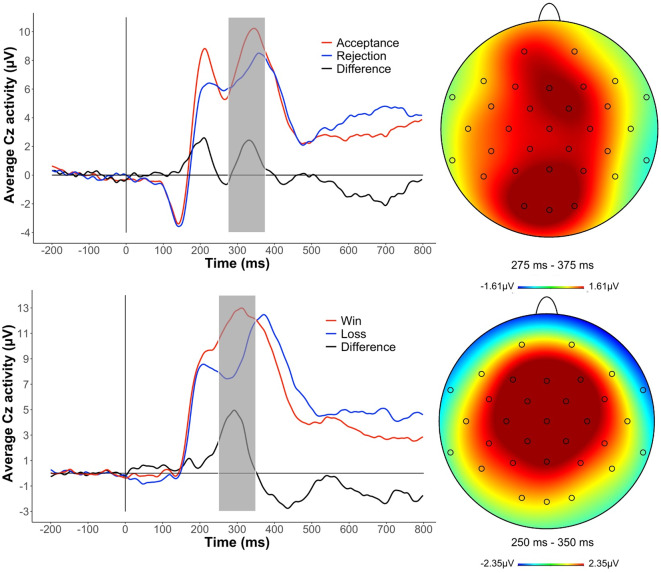
Grand average event-related potential (ERP) waveforms and scalp distributions to social and monetary reward feedback at Cz electrode. Time window is highlighted in gray.

Figure 2 depicts the grand average ERP waveforms and scalp distributions for the two tasks, as well as the time window extracted and used to measure the RewP for each task. As expected, electrocortical responses to rewards (i.e., monetary gains and social acceptance) were greater than those to losses (i.e., monetary losses and social rejection; *t*_(55)_ = 6.056, *p* < 0.001; *t*_(55)_ = 2.802, *p* = 0.007, respectively). Descriptive statistics are presented in Table 1. Early and recent peer victimization were moderately and significantly correlated [Spearman *r* = 0.368, 95% CI = (0.12, 0.58), *p* = 0.007]. Voting behavior and RewP were not significantly correlated [Spearman *r* = 0.003, 95% CI = (−0.27, 0.27), *p* = 0.980]. The results are consistent when outliers (i.e., participants with ERP responses outside 1.5 times the interquartile range) were removed (see Supplemental Materials; Figure S2).

**Table 1:** Descriptive statistics of peer victimization and depression measures.

	Mean	SD	Minimum	Maximum	95% CI for mean
Early HBQ peer victimization	1.43	0.47	1	3.51	(1.31, 1.56)
Recent HBQ peer victimization	1.33	0.46	1	3.2	(1.2, 1.45)
% CDI/BDI items	13.6	12.82	0	55.36	(10.14, 17.07)
N K-SADS MDD symptoms	2.27	2.52	0	9	(1.59, 2.94)

*HBQ, Health and Behavior Questionnaire; CDI, Child Depression Inventory–2; BDI, Beck Depression Inventory–II; KSADS, Kiddie Schedule for Affective Disorders and Schizophrenia; MDD, major depressive disorder*.

### Robust Linear Regressions With Peer Victimization

#### ERP Activity

**Figure 3:**
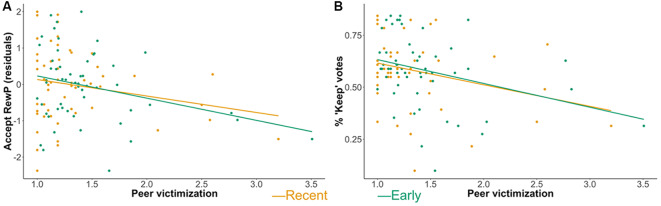
Early and recent peer victimization and **(A)** RewP (residuals) to peer acceptance and **(B)** voting behavior.

Greater early peer victimization was significantly related to a more blunted RewP component to social acceptance [*β* = −0.287, 95% CI = (−0.551, −0.023), *p* = 0.036; see Figure 3A], and remained significant when current age was included as a covariate [*β* = −0.273, 95% CI = (−0.529, −0.018), *p* = 0.039]. Greater recent peer victimization was associated, though not significantly so, with a more blunted RewP component to social acceptance [*β* = −0.207, 95% CI = (−0.473, 0.058), *p* = 0.127; see Figure 3A].

Early and recent peer victimization were not significantly related to the RewP component for monetary gains [*β* = 0.133, 95% CI = (−0.120, 0.386), *p* = 0.297; *β* = 0.184, 95% CI = (−0.049, 0.417), *p* = 0.121, respectively]. When compared, early peer victimization showed a significantly stronger relationship with social rewards than monetary rewards (*Z* = –2.25, *p* = 0.024), as did recent peer victimization (*Z* = –2.17, *p* = 0.030).

The results were consistent when current depression (i.e., CDI/BDI and KSADS) was included as a covariate (see Supplementary Materials).

#### Voting Behavior

Greater early peer victimization was significantly related to fewer votes to accept (i.e., “keep”) other co–players [*β* = −0.325, 95% CI = (−0.606, −0.044), *p* = 0.025; see Figure 3B], and remained significant when current age was included as a covariate [*β* = −0.363, 95% CI = (−0.642, −0.083), *p* = 0.013]. Similarly, greater recent peer victimization was significantly related to fewer votes to accept other co–players [*β* = −0.287, 95% CI = (−0.542, −0.032), *p* = 0.029; see Figure 3B], and remained significant when accounting for current age as a covariate [*β* = −0.288, 95% CI = (−0.557, −0.018), *p* = 0.038]. Results were consistent when current depression (i.e., CDI/BDI and KSADS) was included as a covariate (see Supplementary Materials).

### Robust Linear Regressions With Depression Symptoms

Neither measure of current depression were significantly related to RewP response to social acceptance [CDI/BDI: *β* = −0.078, CI = (−0.370, 0.215), *p* = 0.603; KSADS: *β* = 0.108, CI = (−0.177, 0.392), *p* = 0.463] or voting behavior [CDI/BDI: *β* = 0.024, CI = (−0.247, 0.294), *p* = 0.864; KSADS: *β* = −0.083, CI = (−0.339, −0.173), *p* = 0.522], nor were they significantly related to RewP response to monetary rewards [CDI/BDI: *β* = −0.018, CI = (−0.268, 0.232), *p* = 0.891] [KSADS: *β* = 0.113, CI = (−0.126, 0.353), *p* = 0.354]. Notably, current depression was associated with recent peer victimization, though not significantly [*β* = 0.260, CI = (−0.000, 0.520), *p* = 0.061].

## Discussion

The current study used previously validated social and non-social reward tasks to test the hypothesis that greater peer victimization would be associated with reduced brain responses exclusively to social rewards, whereas greater depression symptoms would be associated with reduced responses to both types of rewards. We found that, among a sample of late-adolescents/young-adults, early and recent peer victimization were related to brain responses to social rewards more so than to monetary rewards, and that greater early experience of peer victimization was related to reduced brain response (i.e., RewP) to peer acceptance. These findings suggest that—as a social stressor—peer victimization is associated with and potentially even shapes the way youth perceive peer interactions and relationships, possibly leading to decreased prosocial behaviors. Research shows that, in children who have experienced victimization, interpersonal skills worsen, attributions of peers become more negative, and they withdraw from or become hostile towards peers (Hymel et al., 1990; Schwartz et al., 1998; Camodeca and Goossens, 2005; Troop-Gordon and Ladd, 2005; Hoglund and Leadbeater, 2007; Bukowski et al., 2010). It is also possible that youth who get less pleasure out of social acceptance are at greater risk of being victimized. In either case, the results speak to the importance of understanding social reward processing throughout development, particularly the consequences of early life social stressors for brain development and behavioral outcomes.

We replicated effects showing that brain responses (i.e., RewP) to rewards were greater than to losses and grand average waveforms largely replicated waveforms from previous studies of both the Island Getaway and Doors tasks (Kujawa et al., 2014, 2017; Proudfit, 2015; Ethridge et al., 2017; Ethridge and Weinberg, 2018). Importantly, the dissociation that peer victimization was associated more strongly with reward response to social acceptance than with monetary rewards suggests that social stresses are linked specifically with deficits in responding to social rewards. Moreover, these results suggest that experience with peer victimization may affect the way social acceptance is represented and valued in the brain—making these experiences less rewarding—rather than leading to generalized blunting to rewards of different types. This emphasizes the importance of incorporating different types of rewards into research on reward-learning and the function of the brain’s reward system. Focusing exclusively on monetary rewards may fail to detect more nuanced investigations of the mechanisms explaining the relationship between psychological stress and psychiatric symptoms. Developmentally, peer relations appear to be salient and rewarding starting in early childhood (Schaefer et al., 2010), suggesting that social rewards do not become salient only in adolescence. In light of this, future studies seeking to characterize deficits in reward function ought to account for different types of rewards.

Additionally, the relationship between peer victimization and reduced acceptance voting indicates that greater victimization is associated with less prosocial behavior, as in other studies of prosocial behavior (Twenge et al., 2007; Di Giunta et al., 2018; Tur-Porcar et al., 2018; Will et al., 2018). This could arise as a socially learned behavior, whereby an adolescent is averse to social acceptance for fear of being rejected in the future. It may also arise as a form of retribution, or getting back at other co-players that did not consistently vote to keep them in the game. On the other hand, voting to reject more often could be interpreted as a strategy for winning the game. This interpretation, however, suggests the possibility that adolescents are using different strategies to win the game: with more victimized youth using a strategy of winning through more “kick out” votes, and less victimized youth using a strategy of accepting other players in the hope they reciprocate. Therefore, whether these individual differences represent affective responses to rejection or a strategy, their behavior is no doubt unlikely to yield greater affiliation with the co-players, and—if taken as an indication of behavior in daily life—unlikely to yield more fulfilling social relationships. Although such reactions could be considered adaptive (i.e., a recently victimized child might reduce the frequency of further victimization by initiating fewer interactions), they are also reducing the overall number of social interactions and thus opportunities for peer acceptance. This could, in turn, increase their vulnerability for depression by making them more isolated and preventing future opportunities for positive social reinforcement. Overall, it appears that recent and early peer victimization biases youth towards more frequent rejection of peers, likely impacting their ability to form interpersonal relationships.

The current study provides further evidence that early life stressors can have consequences for corresponding neural processes and behaviors later in life. Specifically, that a social stressor such as peer victimization can have far-reaching associations with later neural responses and behaviors. The literature on social reward thus far has been primarily focused with adolescence and young adulthood (Casement et al., 2014; Olino et al., 2015; Ethridge et al., 2017, 2018; Kujawa et al., 2017); however our findings suggest that peer victimization may have deleterious effects on youth as early as preschool. Furthermore, they identify possible mediators through which peer victimization is related to depression, or moderators of this relationship. For example, one study suggests a relationship between neural responses to social rejection and depression symptoms in highly victimized girls (Rudolph et al., 2016). Further studies are needed to clarify the causal relationship between peer victimization and depression and to test the role of blunted responding to social rewards and reduced prosocial behavior. Studies that collect information on peer relations and social reward responsivity early in childhood will be of particular importance.

We did not find, in contrast to other studies, that depression was significantly related to neural or behavioral responses to monetary or social rewards (Proudfit, 2015; Nelson et al., 2016; Kujawa et al., 2017). This could be a result of our study being underpowered to detect associations with depression symptoms. Alternatively, depression may be more strongly related with reward anticipation than feedback, in line with some recent fMRI findings (Stoy et al., 2012; Olino et al., 2014; Stringaris et al., 2015; Ubl et al., 2015), and theories that posit a stronger relationship between anhedonia and reward anticipation (Treadway et al., 2012). It is also possible that—in accordance with recent findings suggesting a stronger longitudinal than cross-sectional relationship between monetary reward-responsivity and depression severity (Kujawa et al., 2019)—blunted responses to monetary rewards will predict future depression symptoms. This presents an intriguing future direction to test whether blunted response to social and non-social reward differentially predict future depression severity. Nonetheless, the current findings support the role of a dysfunctional reward system as a neural correlate of social stress, if not also depression.

### Limitations

Despite its strengths, the current study must be considered in light of its limitations. First, although peer victimization was significantly associated with ERP response to social reward and more weakly associated with depression severity, the study may have lacked variability in depression severity needed to detect associations with ERP activation. Second, parent-report of peer victimization was used. The literature suggests that a combination of parent, self, teacher, and peer report is ideal in capturing all aspects of youth’s peer victimization (De Los Reyes and Prinstein, 2004); however, due to study limitations, we were unable to collect these supplementary reports. Third, recent peer victimization was used instead of current peer victimization due to concerns that the nature of victimization would be different once participants were no longer attending high school (i.e., over the age of 18) at the time of assessment, and that parents could be lacking information on their child’s experience with victimization at this age. Fourth, the Doors task does not include a behavioral measure of reward responsivity, limiting our ability to infer how peer victimization is associated with behavioral responses to monetary rewards. Fifth, the RewP is a measure of reward response, accounting for response to losses (i.e., response to monetary gain/social acceptance residualized for response to monetary losses/social rejection) rather than a measure of reward exclusively. A common procedure in ERP research, this is done to isolate activity to the process of interest and account for other overlapping processes. This process does, however, limit the ability to measure the response to reward in isolation or compared to a neutral stimulus. Sixth, despite collecting information on psychotropic medication use in the past year, we did not collect information on medication use during the 48 h prior to the ERP tasks. Seventh, neither task used a measure of reward *learning*. That is, participants’ ability to collect and integrate information to predict a positive outcome. Although the Island Getaway task included a measure of voting behavior, change in trial-to-trial voting was not examined. Future directions to address these limitations are discussed below.

### Future Directions and Conclusions

There are a number of possible future directions to further inform the neural mechanisms underlying the relationship between peer victimization and psychopathology. FMRI studies could inform the location of brain activation linked with peer victimization and examine relationships between peer victimization and reward system network connectivity. Together with the current findings, such studies could identify neural consequences of peer victimization that put individuals at risk for depression. The current study assessed early peer victimization as that occurring between 3 and 7 years of age; future studies could further examine whether this represents a sensitive period. The current study also used an average measure of peer victimization over this period. Studies should seek to further clarify whether it is the chronicity or intensity of peer victimization that is most responsible for blunted reward responses.

Behaviorally, studies using ecological momentary assessment/experience sampling methods (EMA/ESMs) could test whether youth’s behavioral reactions to such laboratory tasks are indeed indicative of their behavior in daily life. Such studies could examine the temporal course of peer victimization, blunted reward response, and behavior, thereby informing the causal relationship between them. Furthermore, other studies using predominately or entirely female samples (89%–100%) have found associations between peer victimization and non-social reward response (Casement et al., 2014; Ethridge et al., 2018). Unfortunately our study was underpowered to assess moderation effects by sex; however, an intriguing direction for future research would be to test whether peer victimization is associated with blunting to rewards in general in females, and more specifically with blunting to social rewards in males.

Finally, the behavioral finding that greater peer victimization is related to fewer acceptance votes appears to be a prime candidate for therapeutic intervention, and speaks to the potential effectiveness of social-emotional interventions aimed at curbing victimized youths’ tendencies to withdraw or lash-out (e.g., Swearer et al., 2017). Further, the blunted neural response may be a particularly useful diagnostic marker, indicating children at especially high risk of developing psychopathology in response to peer victimization. Future studies could additionally incorporate monetary reward tasks that involve a measure of behavior (e.g., monetary incentive delay task) to determine whether peer victimization is also unassociated with behavioral responses to non-social rewards. Overall, the current study emphasizes the meaningful specificity to reward processing of different types. Thus, future research should expand studies of reward processing beyond monetary rewards to account for the possibility that individual differences will be related to other, more domain-specific, types of reward.

## Data Availability

The datasets generated for this study are available on request to the corresponding author.

## Ethics Statement

This study was carried out in accordance with the recommendations of Washington University School of Medicine Institutional Review Board with written informed consent from all subjects. All subjects gave written informed consent in accordance with the Declaration of Helsinki. The protocol was approved by the Washington University School of Medicine Institutional Review Board.

## Author Contributions

BR, LH, AK, JL, and DB were responsible for study concept and design. KA was responsible for coding the Island Getaway task. BR, LH, DK, EK, JL, and DB were responsible for acquisition, analysis, or interpretation of data. BR and DB were responsible for drafting the manuscript and were responsible for statistical analysis. JL and DB obtained funding and supervised the article. BR, KA, DK, JL, and DB were responsible for administrative, technical, or material support. All authors contributed to manuscript revision, read and approved the submitted version.

## Conflict of Interest Statement

JL receives royalties from Guilford Press.
