# Corrigendum: Peer Victimization and Dysfunctional Reward Processing: ERP and Behavioral Responses to Social and Monetary Rewards

**DOI:** 10.3389/fnbeh.2020.00100

**Published:** 2020-06-23

**Authors:** Brent I. Rappaport, Laura Hennefield, Autumn Kujawa, Kodi B. Arfer, Danielle Kelly, Emily S. Kappenman, Joan L. Luby, Deanna M. Barch

**Affiliations:** ^1^Department of Psychological & Brain Sciences, Washington University in St. Louis, St. Louis, MO, United States; ^2^Department of Psychiatry, School of Medicine, Washington University in St. Louis, St. Louis, MO, United States; ^3^Department of Psychology & Human Development, Vanderbilt University, Nashville, TN, United States; ^4^Center for HIV Identification, Prevention, and Treatment Services, University of California, Los Angeles, Los Angeles, CA, United States; ^5^Department of Psychology, San Diego State University, San Diego, CA, United States; ^6^Department of Radiology, School of Medicine, Washington University in St. Louis, St. Louis, MO, United States

**Keywords:** peer victimization, event-related potentials (ERP), reward, depression, adolescence, monetary reward, social reward

In the original article, there was a mistake in Figure 2 as published. In the original article, we had stated that “all data were re-referenced to the average of Tp9 and Tp10” in the Data Analysis section of the Materials and Methods. Although this was our intention, we recently discovered that our scripts had in fact been re-referencing the data to the average of Tp9, Tp10, and Cz electrodes accidentally. Repeating the analyses using the correct referencing and same trials from the original paper yields identical results to those reported in the original article. The corrected Figure 2 appears below. The authors apologize for this error and state that this does not change the scientific conclusions of the article in any way. The original article has been updated.

**Figure 2:**
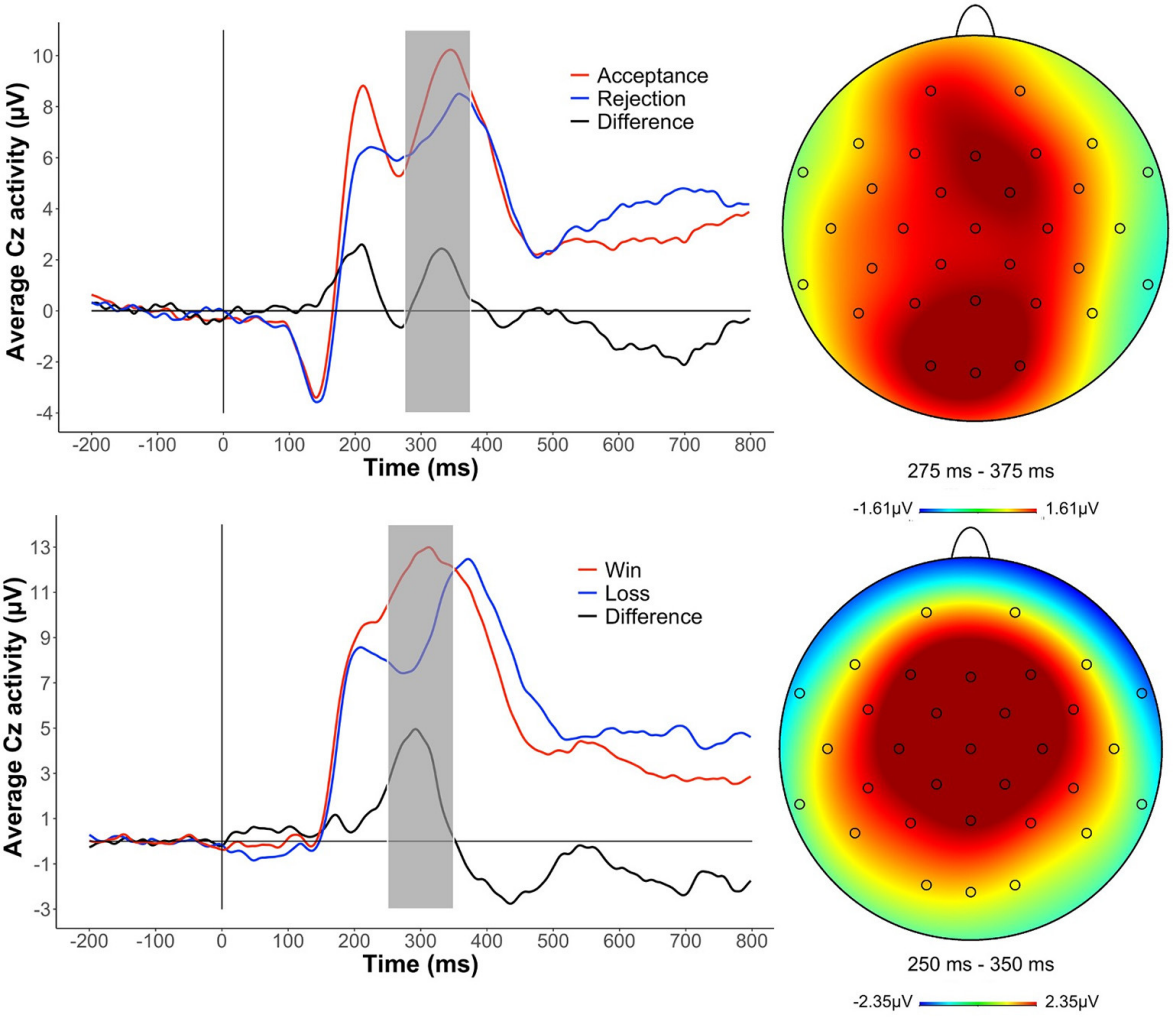
Grand average event-related potential (ERP) waveforms and scalp distributions to social and monetary reward feedback at Cz electrode. Time window is highlighted in gray.

