# Supplementary material for: Peer Victimization and Dysfunctional Reward Processing: ERP and Behavioral Responses to Social and Monetary Rewards
Source: Front Behav Neurosci. 2019 May 31;13:120. doi: 10.3389/fnbeh.2019.00120 (PMC6554678; doi:10.3389/fnbeh.2019.00120)

**Supplemental Material**

Figure S1. Trial during the Doors task.


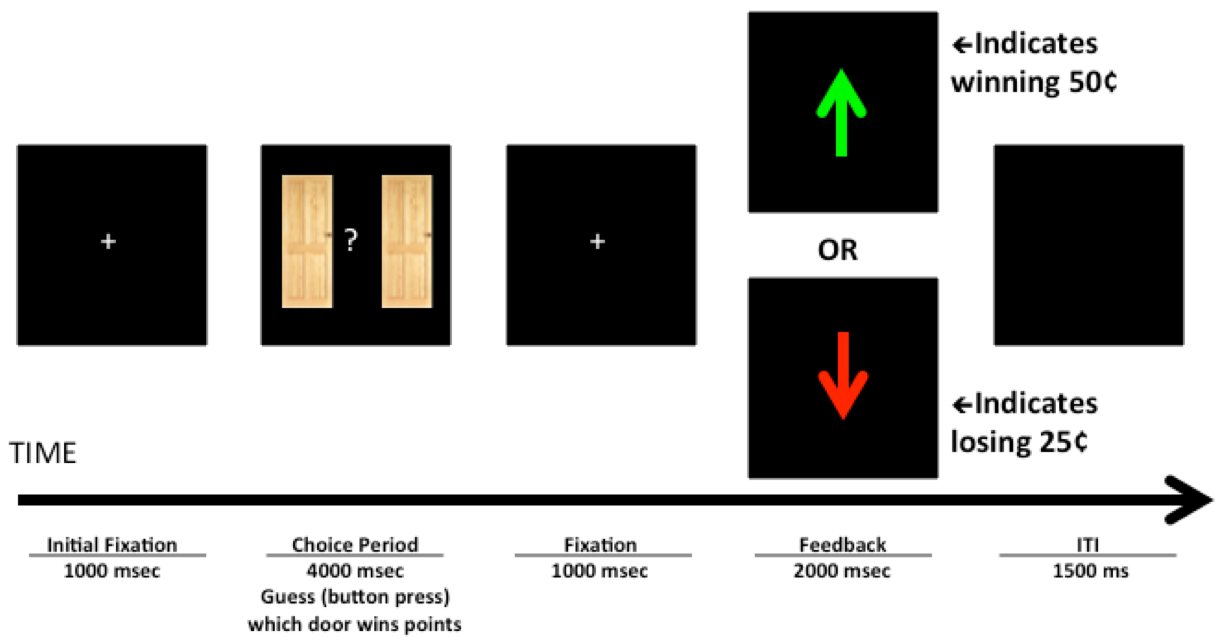


**Supplemental Results**

*Robust linear regressions with physical victimization, rejection, and relational victimization*

*ERP activity*

Greater early rejection (*β* = -0.290, 95% CI = (-0.553, -0.028), *p* = 0.033) was significantly related to a more blunted RewP component to social acceptance, though physical victimization did not (*β* = -0.253, 95% CI = (-0.531, 0.024), *p* = 0.076). Because there is no scale of relational victimization in the HBQ version 1.0, a measure of early relational victimization could not be calculated. Greater recent physical victimization (*β* = -0.167, 95% CI = (-0.434, 0.100), *p* = 0.220), rejection (*β* = -0.199, 95% CI = (-0.467, 0.068), *p* = 0.146), and relational victimization (*β* = -0.198, 95% CI = (-0.461, 0.065), *p* = 0.139) was associated, though not significantly so, with a more blunted RewP component to social acceptance.

*Voting Behavior*

Greater early physical victimization was significantly related to fewer votes to accept (i.e., “keep”) other co–players (*β* = -0.418, 95% CI = (-0.710, -0.125), *p* = 0.007), though early rejection did not (*β* = -0.199, 95% CI = (-0.518, 0.121), *p* = 0.217). Greater recent physical victimization (*β* = -0.323, 95% CI = (-0.526, -0.119), *p* = 0.002) and relational victimization (*β* = -0.294, 95% CI = (-0.549, -0.040), *p* = 0.022) were significantly related to fewer votes to accept other co-players, though recent rejection was not (*β* = -0.175, 95% CI = (-0.455, 0.104), *p* = 0.225).

For the following supplementary analyses, a 250 to 350 ms time window for the Island Getaway task was used in place of the original 275 to 375 ms time window.

*Robust linear regressions with peer victimization*

*ERP activity*

Greater early peer victimization was significantly related to a more blunted RewP component to social acceptance (*β* = - 0.260, 95% CI = (-0.516, -0.003), *p* = 0.048; see Figure S2), and became non-significantly associated when current age was included as a covariate (*β* = -0.244, 95% CI = (-0.513, 0.026), *p* = 0.078). Greater recent peer victimization was associated, though not significantly so, with a more blunted RewP component to social acceptance (*β* = -0.191, 95% CI = (-0.459, 0.078), *p* = 0.162; see Figure S2).

*Robust linear regressions with depression symptoms*

Neither measure of current depression was significantly related to RewP response to social acceptance (CDI/BDI: *β* = - 0.034, CI = (-0.327, 0.258), *p* = 0.818; KSADS: *β* = 0.214, CI = (-0.044, 0.472), *p* = 0.111).

For the following supplementary analyses, outliers with ERP data 1.5 times the interquartile range were removed.

*Robust linear regressions with peer victimization*

*ERP activity*

Greater early peer victimization was related to a more blunted RewP component to social acceptance, nearing significance (*β* = -0.297, 95% CI = (-0.590, -0.004), *p* = 0.051), even when current age was included as a covariate (*β* = -0.286, 95% CI = (-0.567, -0.006), *p* = 0.050). Greater recent peer victimization was associated, though not significantly so, with a more blunted RewP component to social acceptance (*β* = -0.206, 95% CI = (-0.477, 0.065), *p* = 0.139).

Early and recent peer victimization were not significantly related to the RewP component for monetary gains (*β* = 0.204, 95% CI = (–0.102, 0.511), *p* = 0.190; *β* = 0.260, 95% CI = (-0.017, 0.536), *p* = 0.071, respectively). When compared, early peer victimization showed a significantly stronger relationship with social rewards than monetary rewards (Z = -2.32, *p* = 0.020), as did recent peer victimization (Z = -2.36, *p* = 0.019).

*Robust linear regressions with depression symptoms*

Neither measure of current depression was significantly related to RewP response to social acceptance (CDI/BDI: *β* = 0.044, CI = (–0.275, 0.363), *p* = 0.788; KSADS: *β* = 0.195, CI = (-0.086, 0.476), *p* = 0.182) or monetary rewards (CDI/BDI: *β* = 0.143, CI = (-0.161, 0.446), *p* = 0.358; KSADS: *β* = 0.248, CI = (-0.028, 0.525), *p* = 0.083).

For the following supplementary analyses, CDI/BDI and KSADS were included as a covariates

*Robust linear regressions with peer victimization*

*ERP activity*

Greater early peer victimization was significantly related to a more blunted RewP component to social acceptance (*β* = -0.299, 95% CI = (-0.564, -0.033), *p* = 0.029), and remained significant when current age was included as a covariate (*β* = -0.289, 95% CI = (-0.546, -0.033), *p* = 0.029). Greater recent peer victimization was associated, though not significantly so, with a more blunted RewP component to social acceptance (*β* = -0.225, 95% CI = (-0.500, 0.050), *p* = 0.110).

Early and recent peer victimization were not significantly related to the RewP component for monetary gains (*β* = 0.131, 95% CI = (-0.133, 0.396), *p* = 0.321; *β* = 0.199, 95% CI = (-0.054, 0.451), *p* = 0.122, respectively). When compared, early peer victimization showed a significantly stronger relationship with social rewards than monetary rewards (Z = –2.25, *p* = 0.024), as did recent peer victimization (Z = –2.22, *p* = 0.026).

*Voting Behavior*

Greater early peer victimization was significantly related to fewer votes to accept (i.e., “keep”) other co–players (*β* = -0.316 , 95% CI = (-0.609 , -0.022), and remained significant when current age was included as a covariate (*β* = -0.350, 95% CI = (-0.640, -0.060), *p* = 0.021). Similarly, greater recent peer victimization was significantly related to fewer votes to accept other co–players (*β* = -0.302, 95% CI = (-0.578, -0.026), *p* = 0.033), and remained significant when accounting for current age as a covariate (*β* = -0.304, 95% CI = (-0.584, -0.023), *p* = 0.036).

Figure S2. Early and recent peer victimization and RewP (residuals) to peer acceptance, using 250-350 ms time window


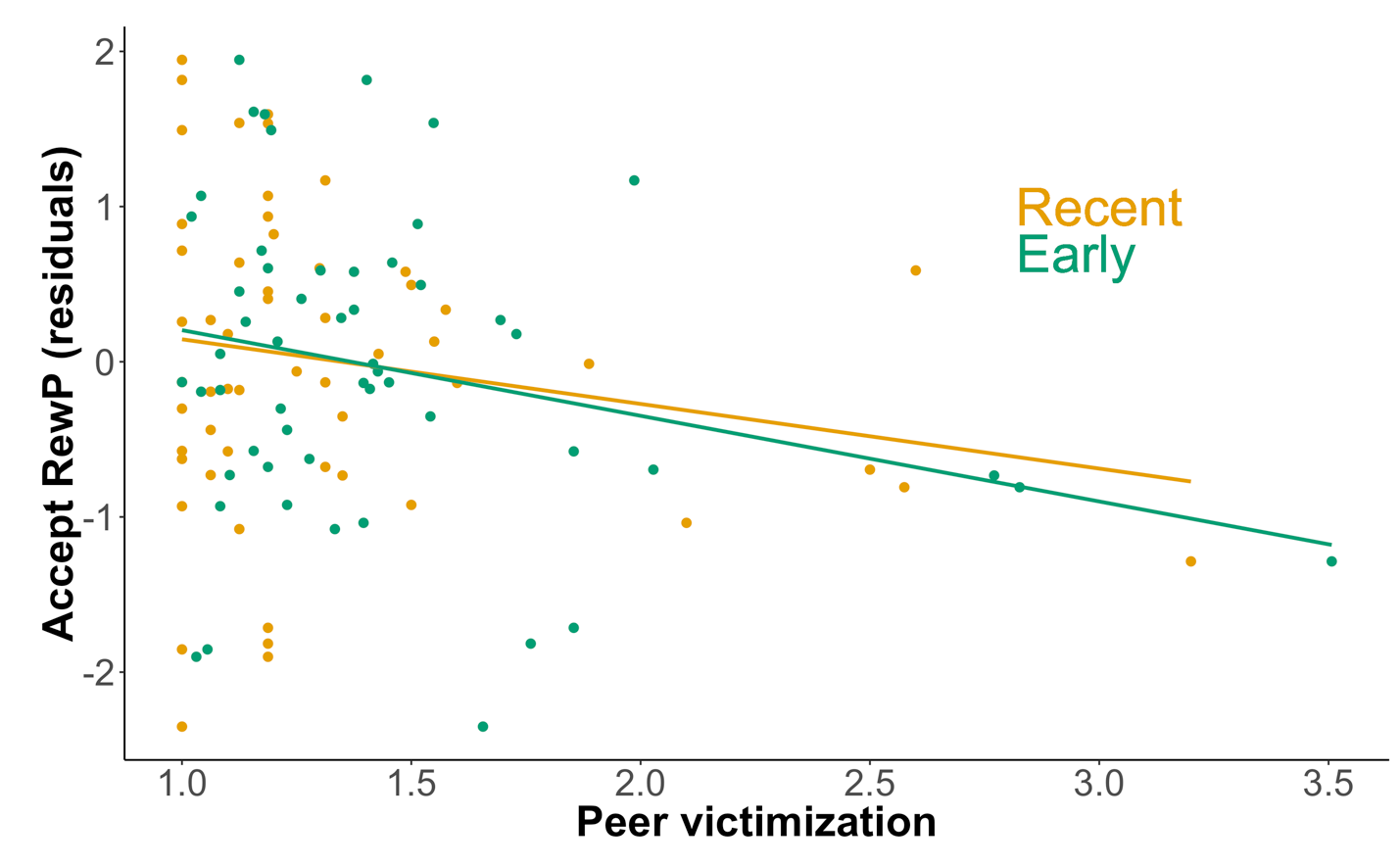

Supplement: Supplementary file 1 [file Data_Sheet_1.docx]
